# Intrafollicular PAI-1 Expression Is Associated with Absence of Histological Transformation During Long-Term Follow-Up in Follicular Lymphoma

**DOI:** 10.3390/ijms27198814

**Published:** 2026-10-01

**Authors:** Cecilie Dohrmann, Marie Hairing Enemark, Caroline Maren Stengaard Jensen, Kristina Lystlund Lauridsen, Trine Lindhardt Plesner, Stephen Jacques Hamilton-Dutoit, Maja Ludvigsen

**Affiliations:** 1 Department of Haematology, Aarhus University Hospital, 8200 Aarhus, Denmark; cecdoh@rm.dk (C.D.); mariem@rm.dk (M.H.E.); caromj@rm.dk (C.M.S.J.); 2 Department of Clinical Medicine, Aarhus University, 8000 Aarhus, Denmark; 3 Department of Pathology, Aarhus University Hospital, 8200 Aarhus, Denmark; krislaur@rm.dk (K.L.L.); stephami@rm.dk (S.J.H.-D.); 4 Department of Pathology, Copenhagen University Hospital, 2100 Copenhagen, Denmark; trine.lindhardt.plesner@regionh.dk

**Keywords:** PAI-1, serpine-1, follicular lymphoma, histological transformation

## Abstract

Follicular lymphoma (FL) is an indolent B-cell malignancy typically associated with favourable outcomes and prolonged survival. However, histological transformation (HT) occurs in a subset of patients and significantly worsens survival outcomes, markedly altering the disease course. Currently, no clinical or molecular predictors of HT are available. We sought to evaluate the potential of Serpin family E member 1 (PAI-1) as a prognostic biomarker of HT in FL, with expression levels assessed by immunohistochemistry and quantified using digital image analysis. PAI-1 expression was evaluated in diagnostic lymphoma biopsies from 30 FL patients with no record of subsequent HT with a minimum follow-up of 10 years, ranging from 10.1 to 24.8 years (non-transforming FL, nt-FL), and 18 patients with subsequent HT occurring 0.7 to 14.6 years after diagnosis (subsequently transforming FL, st-FL); furthermore, the paired high-grade biopsy from the time of HT (transformed FL, tFL) was included. No significant differential expression patterns were identified comparing nt-FL, st-FL and tFL samples. However, high intrafollicular PAI-1 expression at FL diagnosis correlated with no transformation events observed. We believe high intrafollicular PAI-1 expression at presentation might define a minor subgroup of FL patients with reduced risk of HT.

## 1. Introduction

Follicular lymphoma (FL) is the most common indolent B-cell malignancy with an annual incidence of 2–4/100.000 in Western countries [1,2,3]. It is generally considered an incurable disease, with patients often presenting with advanced disease stages [4]. The clinical course of FL often involves several periods of remission interrupted by active disease, including relapses, disease progression, and, in certain cases, the indolent nature of FL transforms into more aggressive lymphoma entities [2,3,4]. Histological transformation (HT) of FL has a cumulative annual risk of 1–3%, predominantly transforming into diffuse large B cell lymphoma (DLBCL) [2,5,6]. The otherwise favourable 5-year overall survival of approximately 90% in FL is significantly altered by this transformation event, declining to 50% upon HT to DLBCL [7,8,9,10]. The risk of HT has been speculated to be influenced by the composition of the tumour microenvironment and the initial choice of therapy [2,11,12,13,14]. However, currently no clinical or biological markers have been demonstrated to be reliably predictive of the transformation event. Furthermore, the underlying biological events leading to HT remain largely unknown. The wide spectrum of disease outcomes and recurrent relapses may pose a significant challenge to patients’ psychological well-being and highlights the need for more clearly defined personalized risk assessments at diagnosis [15]. Our group previously identified differential mRNA expression of *SERPINE1* in diagnostic and transformed FL biopsies. Higher *SERPINE1* expression was observed in diagnostic biopsies from FL patients experiencing subsequent transformation [13]. The encoded protein, Serpin family E member 1 (PAI-1), is a serine protease inhibitor that targets tissue-type plasminogen activator (tPA) and urokinase-type plasminogen activator (uPA). Inhibition of tPA primarily suppresses fibrinolysis, whereas inhibition of uPA affects cytoskeletal reorganization, extracellular matrix (ECM) degradation and cell motility. The serine protease uPA converts plasminogen to plasmin, thereby promoting ECM degradation and activation of matrix metalloproteinases, ultimately facilitating cellular migration [16,17,18]. Elevated tumour levels of uPA-encoding *PLAU* mRNA have previously been associated with poor clinical outcomes in other solid cancers [19,20]. In the present study, we assessed PAI-1 protein expression in diagnostic biopsies from FL patients with no subsequent HT within a minimum of 10 years of follow-up (nt-FL) and in patients who subsequently transformed, both in their diagnostic FL biopsy (st-FL) and the matched lymphoma biopsy obtained at the time of transformation (tFL). Associations between tumour tissue PAI-1 expression and clinicopathological features, subsequent transformation status, and transformation-free survival were investigated.

## 2. Results

**Figure 1:**
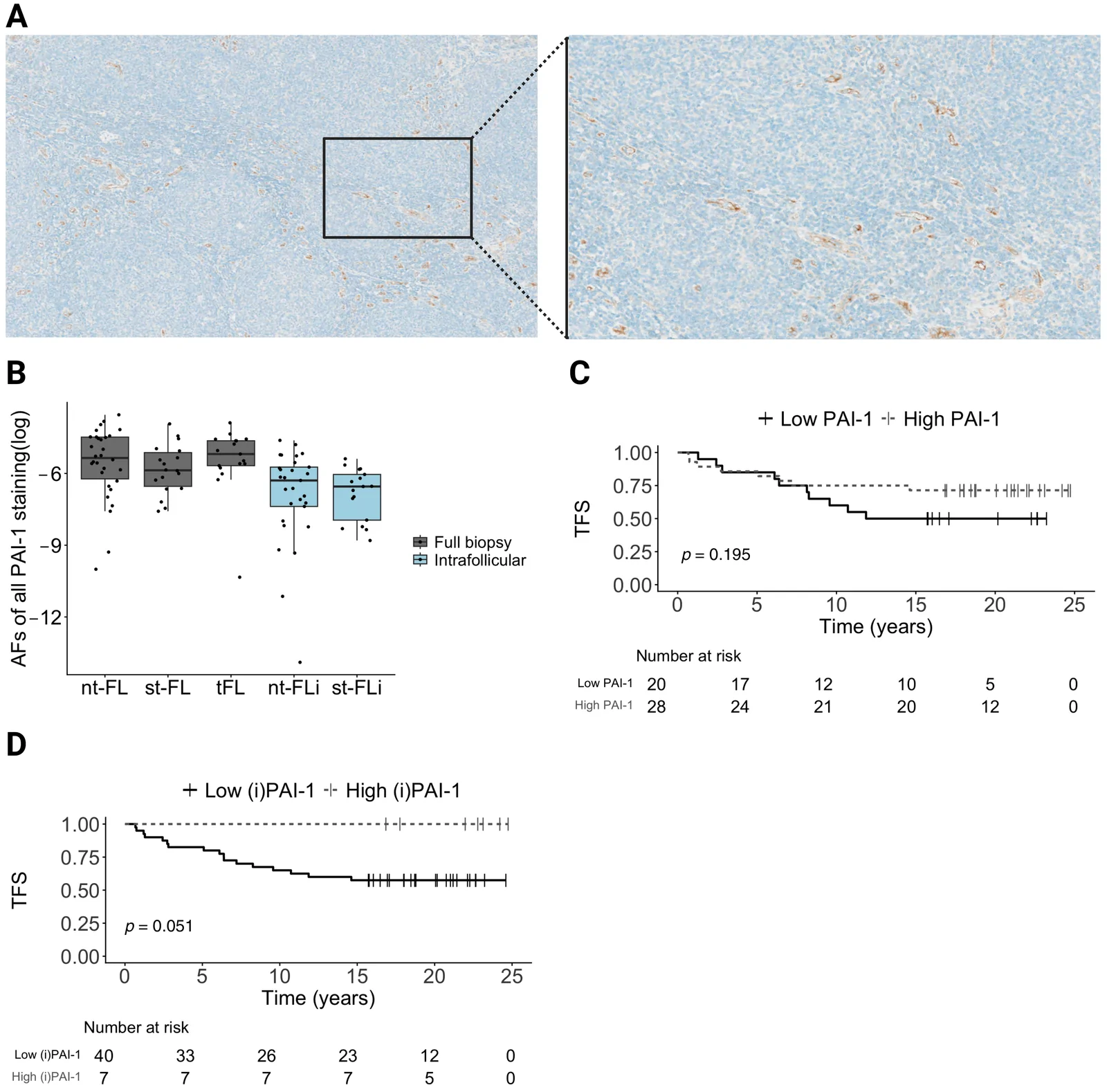
Tumoral expression of PAI-1 in FL and its association with TFS. (**A**) Immunohistochemical staining for PAI-1 in a diagnostic biopsy. Left: magnification at 5×, right: magnification at 10×. (**B**) Boxplot showing AFs of PAI-1 expression levels in full biopsy (grey) and intrafollicular areas (blue) in nt-FL, st-FL and tFL samples. No significant differences were observed. (**C**) Kaplan–Meier curves for TFS stratified by full biopsy PAI-1 expression (cutoff AF = 0.0029). (**D**) Kaplan–Meier curves for TFS stratified by intrafollicular PAI-1 expression (cutoff AF = 0.0046). Abbreviations: AF, area fraction; nt-FL, non-transforming FL; i, intrafollicular; PAI-1, Serpin family E member 1; st-FL, subsequently transforming FL; tFL, transformed FL; TFS, transformation-free survival.

The cohort comprised 23 males and 25 females, with a median age at diagnosis of 53.5 years (range 35–76), as shown in Table 1. Among them, 30 were non-transforming patients (nt-FL) and 18 were subsequently transforming patients (st-FL). For st-FL patients, a paired transformed biopsy (tFL) was included. The clinicopathological characteristics of the two diagnostic groups were similar, although st-FL presented with more advanced Ann Arbor stages and higher FLIPI scores. Patients in the nt-FL group were followed for a mean of 19 years, ranging from 10.1 to 24.8 years. All transformation events in the st-FL group occurred in the range of 0.7 to 14.6 years after FL diagnosis. The immunohistochemical staining of PAI-1 in the lymphoma biopsies was primarily detected in the cytoplasm of extrafollicular cells and endothelial cells, and was furthermore present within the vascular lumen, as shown in Figure 1A. Overall low-intensity PAI-1 expression was identified in all diagnostic biopsies with a tumour tissue median area fraction (AF) of 0.393% (range, 0.005–2.880%). We found no statistically significant difference in expression levels between nt-FL (median 0.474%, range 0.005–2.880%) and st-FL (median 0.283%, range 0.051–1.970%) samples. Also, no significant changes were observed in the tFL samples (median 0.558%, range 0.003–2.060%), as shown in Figure 1B. Since PAI-1 expression was found primarily in the tumour microenvironment rather than in the malignant B cells that are primarily located in the follicular structures, we also focused our analysis on intrafollicular areas to assess microenvironmental PAI-1 expression in close proximity to the malignant B cells. An analogous pattern was evident in intrafollicular areas (nt-FL, median 0.196%, range <0.001–0.989%; st-FL, median 0.144%, range 0.015–0.458%), as shown in Figure 1B. Furthermore, PAI-1 expression was not significantly associated with any of the presented clinicopathological features, as shown in Table S1. Based on the hypothesis that crosstalk between the tumour microenvironment and malignant B cells contributes to the underlying biological mechanisms of transformation, we examined the correlation between PAI-1 expression and the expression of previously characterized markers as a potential indicator of the interplay in the FL tumour microenvironment.

Tumoral expression of PAI-1 positively correlated with the expression of IDO1 (rho = 0.340, *p* = 0.020), which was previously identified by our group [21]. Under inflammatory conditions, IDO1 can also be expressed by endothelial cells, and our previous study indicated that IDO1 expression in FL is primarily localized to the extrafollicular regions of the tumour microenvironment, consistent with the expression patterns observed in the immunohistochemical evaluation of PAI-1. Furthermore, three other markers previously investigated by our group correlated with PAI-1 expression: BAX (rho = 0.337, *p* = 0.019), SEPT6 (rho = 0.386, *p* = 0.007), and CD81 (rho = 0.586, *p* = 7.778 × 10^−5^) [22,23]. SEPT6 and CD81 have, similar to PAI-1, been implicated in cell adhesion, migration and cytoskeletal reorganization [23,24]. Migration, adhesion and cellular survival are interconnected cellular processes, and beyond PAI-1’s primary role in migration and invasion, the PAI-1 and uPA interplay has been reported to affect survival signalling pathways, potentially accounting for the observed correlation with the proapoptotic molecule BAX [25,26]. However, to the best of our knowledge, no direct well-established biological interplay has been reported between PAI-1 and the presented proteins, as shown in Table S1. Evaluating AFs of PAI-1 expression in whole lymphoma biopsy specimens revealed no significant association between PAI-1 expression and transformation-free survival (TFS; *p* = 0.195, cutoff AF = 0.0029), as shown in Figure 1C, but when restricting the analysis to intrafollicular areas only, high levels of PAI-1 revealed borderline significant associations with superior transformation-free survival (TFS; *p* = 0.051, cutoff AF = 0.0046), as shown in Figure 1D. Notably, none of the patients classified as having high intrafollicular PAI-1 expression underwent transformation, while 17 patients, classified as having low intrafollicular PAI-1 expression at diagnosis, experienced transformation. Hence, based on intrafollicular PAI-1 protein expression at the time of diagnosis, we were able to identify a patient subgroup that did not undergo HT during the long follow-up period.

## 3. Discussion

Utilizing immunohistochemistry (IHC) followed by digital image analysis, we performed a retrospective observational study on archival FL biopsies and assessed the protein expression levels of PAI-1 in diagnostic and transformed FL biopsies. The key finding of this study was the association between high intrafollicular PAI-1 expression at diagnosis and subsequent HT, where no patients in this group experienced HT during long-term follow-up. Low levels of intrafollicular PAI-1 were associated with shorter TFS, which is biologically plausible given its role as an inhibitor of uPA and thus inhibition of ECM degradation and cellular migration. In this context, higher PAI-1 levels may be associated with more favourable clinical outcomes by limiting uPA-mediated invasion and disease dissemination. To the best of our knowledge, no previous studies have examined PAI-1 in FL. However, PAI-1 has previously been investigated in a considerable number of other cancer types. In several solid cancers, high levels of PAI-1 have been associated with a poorer prognosis, and pre-clinical studies have investigated the role of PAI-1 inhibition in solid malignancies. Despite the negative prognostic role of PAI-1 in several human cancer types, the therapeutic effects of PAI-1 inhibition are still inconclusive [27,28,29,30,31]. A large pan-cancer study investigated the prognostic value of *SERPINE1* gene expression, and the outcome included both protective and detrimental roles [32]. Multiple mechanisms by which PAI-1 may contribute to cancer progression have been proposed, including roles in cell cycle regulation, angiogenesis, inhibition of apoptosis, cell migration, and immune modulation [18,33,34]. These multifaced roles of PAI-1 in cancer are referred to as the “PAI paradox”, and the diverse biological functions could explain the varying prognostic significance reported across different cancer types; elucidating the precise molecular mechanisms of PAI-1 in FL and other cancer types may be of considerable relevance to understanding its prognostic impact [25]. Furthermore, mapping the precise cellular origins of PAI-1 in FL may improve our understanding of its potential prognostic relevance. Previously, we identified PAI-1-encoding *SERPINE1* mRNA as differentially expressed between st-FL and nt-FL patients (fold change 2.99, *p* < 0.001) by bulk NanoString transcript expression array. Moreover, *SERPINE1* mRNA was further upregulated in paired tFL samples compared with st-FL samples (fold change 1.97, *p* = 0.008) [13]. In the present study focusing on protein expression, we did not detect significant intratumoral differences in PAI-1 protein expression levels between nt-FL and st-FL samples. Although not statistically significant, st-FL exhibited lower PAI-1 levels than nt-FL, trending in the opposite direction to the pattern observed in our gene expression data [13]. The same pattern was seen in the intrafollicular analysis. The gene expression analysis of *SERPINE1* mRNA levels was not reflected in the immunohistochemical evaluation, and this discrepancy may reflect methodological differences and limited concordance between mRNA and the actual protein abundance. Expression levels varied considerably between patients, and the limited cohort size of this study should be considered when interpreting the results presented, especially given the heterogeneity of FL patients. Furthermore, the gene expression analysis was performed on bulk tumour tissue, including adipose and connective tissue, whereas the present study was restricted to lymphoid tissue. The tumour tissue of FL patients exhibits considerable PAI-1 expression heterogeneity, which may reflect differences in the cellular composition of the tumour, and since PAI-1 is primarily expressed in endothelial cells, the PAI-1 pattern may represent levels of microvessels inside the lymph node. The number of microvessels has previously been reported to be associated with clinical outcomes in FL; however, most findings indicate that high microvessel density is associated with inferior prognosis [35,36,37]. Additionally, posttranslational regulation, including translation efficiency, protein stability and temporal differences, may explain the diverging findings in mRNA and protein levels.

When interpreting these findings, the hypothesis-generating nature of the present study should be considered, as the AF cutoff value used to dichotomise the PAI-1 expression levels was data-driven, potentially introducing overfitting. Furthermore, differences in baseline characteristics and therapeutic regimens may confound the observed association between diagnostic expression levels of PAI-1 and the subsequent transformation status.

Still, the main finding of this study indicates that a high diagnostic level of intrafollicular PAI-1 is associated with reduced risk of subsequent transformation. However, the implications of this for introducing novel therapeutic strategies in FL are not straightforward, since treatment interventions associated with protein upregulation are typically complex and associated with the risk of deregulation of physiological PAI-1 functions. In addition, predicting the outcome of therapeutic intervention is complex given the broad biological activities of PAI-1. Thus, PAI-1 might not be an ideal therapeutic target in FL. Nonetheless, the potential prognostic value of diagnostic PAI-1 expression for subsequent HT is of particular importance in a cancer such as FL in which adequate individualized risk stratification is currently lacking, and where identifying true low-risk patients may improve quality of life for a substantial proportion of the patient population. Thus, upfront immunohistochemical identification of PAI-1 expression in FL specimens may present considerable potential for simple and cost-effective implementation in the clinical routine at pathology departments to assist in identification of FL patients with a low risk of HT. However, the exploratory nature of this study demands validation in larger independent cohorts before considering implementation of PAI-1 expression as a prognostic marker of transformation in FL.

## 4. Materials and Methods

To evaluate PAI-1 protein expression, 48 diagnostic formalin-fixed, paraffin-embedded lymphoma biopsies were analysed by IHC. The biopsies were all reviewed by expert haematopathologists, and diagnostic and transformed biopsies were classified according to the World Health Organization 2022 classification criteria for lymphoid tumours [38]. The cohort has previously been described [21,22,23,39,40,41,42]. All patients were diagnosed with FL grade 1–3A between 1990 and 2015 at the Department of Haematology, Aarhus University Hospital. From the diagnostic FL samples, 18 patients subsequently presented with transformation into FL grade 3B or DLBCL. Paired high-grade tFL samples obtained at HT were also included, and only patients presenting with HT more than six months after the date of diagnosis were included in this study. This threshold was applied to exclude composite and discordant lymphomas from this cohort. The remaining 30 patients showed no evidence of HT; for these patients, a minimum follow-up period of ten years was required. Patients' clinicopathological data were obtained from the Danish Lymphoma Registry [43]. Treatment records were only available for a limited subset of the cohort and included alkylating agent-based, anthracycline-based and rituximab-containing treatment regimens. IHC staining was performed utilizing an automated staining system, Ventana Benchmark Ultra (Ventana Medical systems, Roche, Tucson, AZ, USA), as previously described [21,22,23,39,40,41,42]. The IHC staining platform utilized heat-induced epitope retrieval for 92 min at 100 °C. The primary antibody anti-PAI-1 (HPA050039, Sigma Aldrich, St. Louis, MO, USA) was added at a 1:100 dilution, followed by a 32 min. incubation at 37 °C. Visualisation was performed using the OptiView DAB Detection Kit (Ventana, 760–700) and counterstaining of cell nuclei was obtained utilizing haematoxylin. Sections of appendix, tonsil, liver and pancreas were included as positive and negative controls. Slides were scanned on the NanoZoomer 2.0 HT (Hamamatsu Photonics, Hamamatsu, Shizuoka, Japan) and analysed in Visiopharm 2024.07 (Visiopharm A/S, Hoersholm, Denmark). The analysis output was defined as AFs of positive PAI-1 staining relative to either the total lymphoid area or the area of included follicular structures. Areas of non-lymphoid tissue and technical artifacts were excluded from the analysis, and intrafollicular areas were manually delineated using consecutive PAX-5-stained sections. Statistical analyses were performed in RStudio (version 4.2.2), and the cutoff values utilized for dichotomous biomarker expression were based on Youden’s index and ROC analysis. ROC curve analysis was performed using subsequent transformation status as the endpoint. Detailed descriptions of materials and methods are available in the Supplementary Materials.

## 5. Conclusions

In conclusion, we report an overall low intensity PAI-1 expression in both diagnostic and transformed FL biopsies, providing a rationale for further investigation of PAI-1 as a potential prognostic marker in FL. We found that tumours with comparatively higher intrafollicular PAI-1 expression were associated with a FL subgroup with no observed HT during long-term follow-up. Although this finding requires validation in larger independent cohorts, it suggests that it may be possible to identify a patient subgroup with a reduced risk of transformation already at the time of diagnosis.

## Figures and Tables

**Table 1:** Patients’ clinicopathological features.

Characteristics	All *n* = 48 *n* (%)	nt-FL *n* = 30 *n* (%)	st-FL *n* = 18 *n* (%)	*p*-Value
Sex				NS
Male	23 (58)	13 (43)	10 (56)	
Female	25 (42)	17 (57)	8 (44)	
Age at FL diagnosis				NS
Median	53.5	53.5	53	
Range	35–76	35–76	40–74	
Ann Arbor stage				0.008
I-II	15 (31)	14 (47)	1 (5)	
III-IV	31 (65)	15 (50)	16 (89)	
Unknown	2 (4)	1 (3)	1 (5)	
FLIPI				0.012
Low (0–1)	19 (40)	16 (53)	3 (17)	
Intermediate (2–3)	23 (48)	12 (40)	11 (61)	
High (4–5)	2 (4)	0 (0)	2 (11)	
Unknown	4 (8)	2 (7)	2 (11)	
B-symptoms				NS
Yes	11 (23)	5 (17)	6 (33)	
No	34 (71)	24 (80)	10 (56)	
Unknown	3 (6)	1 (3)	2 (11)	
LDH-elevation				NS
Yes	2 (4)	1 (3)	1 (6)	
No	42 (88)	27 (90)	15 (83)	
Unknown	4 (8)	2 (7)	2 (11)	
Performance score				NS
≤2	45 (94)	29 (97)	16 (89)	
>2	0 (0)	0 (0)	0 (0)	
Unknown	3 (6)	1 (3)	2 (11)	
Anaemia				NS
Yes	4 (8)	1 (3)	3 (17)	
No	41 (86)	28 (94)	13 (72)	
Unknown	3 (6)	1 (3)	2 (11)	
FL histology				NS
Grade 1–2	41 (85)	25 (83)	16 (89)	
Grade 3A	7 (15)	5 (17)	2 (11)	

LDH, lactate dehydrogenase; FLIPI, Follicular Lymphoma International Prognostic Index; nt-FL, non-transforming FL; NS, not significant; st-FL, subsequently transforming FL.

## Data Availability

Data will be available upon reasonable request to the corresponding author.

## Supplementary Materials

The following supporting information can be downloaded at: https://www.mdpi.com/article/10.3390/ijms27198814/s1.
