# Supplementary material for: Intrafollicular PAI-1 Expression Is Associated with Absence of Histological Transformation During Long-Term Follow-Up in Follicular Lymphoma
Source: Int J Mol Sci. 2026 Oct 1;27(19):8814. doi: 10.3390/ijms27198814 (PMC13665162; doi:10.3390/ijms27198814)
Supplement: Supplementary file 1 [file ijms-27-08814-s001.zip › ijms-4554394-supplementary.pdf]

Supplementary Methods

Patient cohort

Immunohistochemical evaluation was performed on pre-therapeutic formalin-fixed, paraffin embedded (FFPE) tumour tissue specimens from a cohort of 48 FL patients diagnosed with FL grade 1-3A at Aarhus University Hospital, Denmark, between 1990 and 2015. The cohort included 30 patients with no record of transformation within a minimum of 10 years of follow-up (non-transforming FL, nt-FL) and 18 patients with subsequent histological transformation (HT) to DLBCL or FL grade 3B, at least 6 months after the primary FL diagnosis (subsequently transforming FL, st-FL). Furthermore, for the 18 st-FL patients, paired transformed lymphoma samples from the time of histological transformation (HT) were also analysed (histologically transformed FL, tFL). All biopsies were reviewed and classified according to the 2022 update on the WHO Classification of Tumours of the Haematopoietic and Lymphoid Tissues by two experienced hematopathologists (SJHD and TLP) [1]. The patients´ clinicopathological data were obtained from the Danish Lymphoma Registry [2]. The clinicopathological data of this cohort and immunohistochemical data on other putative biological markers have been published previously [3-9]. This study was approved by the Danish National Committee on Health Research Ethics (1-10-72-276-13, date of most recent approval 02.03.2026) and the Danish Data Protection Agency (1-16-02-407-13, date of most recent approval 29.01.2026) and was conducted in accordance with the Declaration of Helsinki.

Immunohistochemical staining for PAI-1

Immunohistochemical staining was performed on 4µm FFPE sections using the Ventana Benchmark Ultra automated staining system (Ventana Medical Systems, Tucson, AZ) using standard methods. Slides were deparaffinized followed by blocking of endogenous peroxidase activity using the OptiView DAB IHC Detection Kit (Ventana, 760-700). Heat induced epitope retrieval was applied at 100ºC for 92 minutes in ULTRA Cell Conditioning Solution 1 (Ventana, 950-224). Primary polyclonal anti-human anti-PAI-1 primary antibody (HPA050039, Sigma-Aldrich, MO) was diluted 1:100 in Tris buffered antibody diluent (pH 7.2, 15mmol/L NaN3 and stabilizing protein, Dako, Santa Clara, CA) followed by 32 minutes of incubation at 37°C. Visualization was performed using the OptiView DAB IHC Detection Kit (Ventana, 760-700) and the specimens were counterstained by hematoxylin. Sections of appendix, tonsil, liver, and pancreas were included on all slides as positive and negative controls [3-9].

Digital image analysis

Stained slides were scanned at a magnification of x20 using the Hamamatsu Nanozoomer 2.0HT scanner (Hamamatsu, Shizouka, Japan). Expression levels of PAI-1 were quantified using Visiopharm Integrator system (Visiopharm A/S, Hoersholm, Denmark). Areas of lymphoid tissue suitable for staining quantification were defined by manual outlining of regionss of interest (ROI) on each digitized section. Distinct areas of non-lymphoid tissue and technical artefacts were excluded. Intrafollicular areas were manually outlined using a consecutive PAX5-stained section to identify follicular areas. One slide was excluded due to inability to reliably identify intrafollicular regions. All samples were assessed by a single observer. The observer was not blinded to transformation status. An analysis protocol package (APP) was

designed to quantify the expression levels. Staining quantification outputs were area fractions (AFs), defined as the stained area normalized to the total area within the ROI. Expression levels were calculated on AFs of all positive staining [3-9].

Statistical analysis

Differences in clinicopathological features were assessed using either the Chi-squared test or Fisher's exact test, depending on whether the test assumptions were satisfied. Analyses were performed using available clinical data, and missing data were not imputed. Correlation of PAI-1 expression with previously investigated markers and clinicopathological features was evaluated using a Spearman's rank test. Due to the exploratory nature of the study, multiple testing correction was not applied. Differences in AFs from nt-FL, st-FL, and tFL samples were assessed using an independent Mann-Whitney U test and a paired Wilcoxon ranked sum test. Time-related endpoints were analysed using the Kaplan Meier and log rank method with the endpoint transformation-free survival (TFS). TFS was defined as time from initial FL diagnosis to the date of biopsy-proven HT or censoring. Cutoff values for high versus low PAI-1 expression for TFS analyses were determined by a ROC analysis with the optimal cutoff point calculated using Youden's index. The PAI-1 cutoff values were empirically determined based on the expression data in the present cohort with the endpoint being subsequent transformation. The AF cutoff value in the full biopsy was 0.0029. At this cut point the sensitivity and specificity was 55.6 (95% CI 33.7-75.4%) and 70% (95% CI 52.1-83.3%) respectively. The area under the ROC curve was 0.611(95% CI 0.447-0.775). In the intrafollicular analysis the AF cutoff value was 0.0046. At this cut point the sensitivity and specificity was 100% (95% CI 81.6-100%) and 23.3% (95% CI 11.8-40.9%) respectively. The area under the ROC curve was 0.575 (95% CI 0.410-0.739). Due to the limited number of transformation events, Cox regression analyses were not performed, and hazard ratios were therefore not estimated. P-values below 0.05 were considered statistically significant. Statistical analyses were performed using R Statistical Software (2026.01.1+403) [3-9].

Supplementary Table

	**PAI-1**
**Sex**	NS
**Age at FL-diagnose**	NS
**Ann Abor stage**	NS
**FLIPI**	NS
**B-symptoms**	NS
**LDH-elevation**	NS
**Performance score**	NS
**Anaemia**	NS
**FL-grade**	NS
**Nodal sites**	NS
**SEPT6**	p = 0.007
**BAX**	p = 0.019
**CD81**	p < 0.001
**IDO1**	p = 0.020

Table S1: Correlation of PAI-1 expression with clinicopathological features and other previously investigated FL-markers: Abbreviations: FLIPI, follicular lymphoma international prognostic index**.** LDH, lactate dehydrogenase. NS, non-significant. SEPT6, Septin 6. BAX, BCL2 Associated X. CD81, CD81 Molecule. IDO1, Indoleamine 2,3-Dioxygenase 1.

Supplementary References

1. Alaggio, R.; Amador, C.; Anagnostopoulos, I.; Attygalle, A.D.; Araujo, I.B.O.; Berti, E.; Bhagat, G.; Borges, A.M.; Boyer, D.; Calaminici, M.; et al. The 5th edition of the World Health Organization Classification of Haematolymphoid Tumours: Lymphoid Neoplasms. *Leukemia* **2022**, *36*, 1720–1748, doi:10.1038/s41375-022-01620-2.
2. Arboe, B.; El-Galaly, T.C.; Clausen, M.R.; Munksgaard, P.S.; Stoltenberg, D.; Nygaard, M.K.; Klausen, T.W.; Christensen, J.H.; Gørløv, J.S.; Brown, P.d.N. The Danish National Lymphoma Registry: Coverage and Data Quality. *PLOS ONE* **2016**, *11*, e0157999, doi:10.1371/journal.pone.0157999.
3. Enemark, M.B.H.; Wolter, K.; Campbell, A.J.; Andersen, M.D.; Sørensen, E.F.; Hybel, T.E.; Madsen, C.; Lauridsen, K.L.; Plesner, T.L.; Hamilton-Dutoit, S.J.; et al. Proteomics identifies apoptotic markers as predictors of histological transformation in patients with follicular lymphoma. *Blood Adv* **2023**, *7*, 7418–7432, doi:10.1182/bloodadvances.2023011299.
4. Enemark, M.H.; Wolter, K.; Hybel, T.E.; Andersen, M.D.; Sørensen, E.F.; Hindkaer, L.M.; Lauridsen, K.L.; Madsen, C.; Plesner, T.L.; Hamilton-Dutoit, S.; et al. Differential tumor protein expression at follicular lymphoma diagnosis reveals dysregulation of key molecular pathways associated with histological transformation. *Scientific Reports* **2024**, *14*, 29962, doi:10.1038/s41598-024-81693-4.
5. Enemark, M.B.H.; Sørensen, E.F.; Hybel, T.E.; Andersen, M.D.; Madsen, C.; Lauridsen, K.L.; Honoré, B.; d'Amore, F.; Plesner, T.L.; Hamilton-Dutoit, S.J.; et al. IDO1 Protein Is Expressed in Diagnostic Biopsies from Both Follicular and Transformed Follicular Patients. *International Journal of Molecular Sciences* **2023**, *24*, 7314.
6. Enemark, M.B.; Hybel, T.E.; Madsen, C.; Lauridsen, K.L.; Honoré, B.; Plesner, T.L.; Hamilton-Dutoit, S.; d'Amore, F.; Ludvigsen, M. Tumor-Tissue Expression of the Hyaluronic Acid Receptor RHAMM Predicts Histological Transformation in Follicular Lymphoma Patients. *Cancers* **2022**, *14*, 1316.
7. Monrad, I.; Madsen, C.; Lauridsen, K.L.; Honoré, B.; Plesner, T.L.; Hamilton-Dutoit, S.; d'Amore, F.; Ludvigsen, M. Glycolytic biomarkers predict transformation in patients with follicular lymphoma. *PLOS ONE* **2020**, *15*, e0233449, doi:10.1371/journal.pone.0233449.
8. Madsen, C.; Lauridsen, K.L.; Plesner, T.L.; Monrad, I.; Honoré, B.; Hamilton-Dutoit, S.; d'Amore, F.; Ludvigsen, M. High intratumoral expression of vimentin predicts histological transformation in patients with follicular lymphoma. *Blood Cancer J* **2019**, *9*, 35, doi:10.1038/s41408-019-0197-5.
9. Beck Enemark, M.; Monrad, I.; Madsen, C.; Lystlund Lauridsen, K.; Honoré, B.; Plesner, T.L.; Hamilton-Dutoit, S.J.; d'Amore, F.; Ludvigsen, M. PD-1 Expression in Pre-Treatment Follicular Lymphoma Predicts the Risk of Subsequent High-Grade Transformation. *Onco Targets Ther* **2021**, *14*, 481–489, doi:10.2147/ott.S289337.
